# Chronic Heat Stress Caused Lipid Metabolism Disorder and Tissue Injury in the Liver of *Huso dauricus* via Oxidative-Stress-Mediated Ferroptosis

**DOI:** 10.3390/antiox14080926

**Published:** 2025-07-29

**Authors:** Yining Zhang, Yutao Li, Ruoyu Wang, Sihan Wang, Bo Sun, Dingchen Cao, Zhipeng Sun, Weihua Lv, Bo Ma, Ying Zhang

**Affiliations:** 1 Key Laboratory of Cold Water Fish Germplasm Resources and Multiplication and Cultivation of Heilongjiang Province, Heilongjiang River Fishery Research Institute, Chinese Academy of Fishery Sciences, Harbin 150070, China; zhangyining@hrfri.ac.cn (Y.Z.); liyutao@neau.edu.cn (Y.L.); wangsihan@hrfri.ac.cn (S.W.); sunbo@hrfri.ac.cn (B.S.); caodingchen@hrfri.ac.cn (D.C.); sunzhipeng@hrfri.ac.cn (Z.S.); lvweihua@hrfri.ac.cn (W.L.); 2 College of Fisheries and Life Science, Shanghai Ocean University, Shanghai 201306, China; mabo@hrfri.ac.cn; 3 Heilongjiang River Basin Fishery Resources and Environment Scientific Observation Experimental Station, Heilongjiang River Fishery Research Institute, Chinese Academy of Fishery Sciences, Harbin 150070, China

**Keywords:** *Huso dauricus*, oxidative stress, heat stress, liver, transcriptome, kaluga sturgeon

## Abstract

High-temperature stress has become an important factor that has restricted the aquaculture industry. *Huso dauricus* is a high-economic-value fish that has faced the threat of thermal stress. Based on this point, our investigation aimed to explore the detailed mechanism of the negative impacts of heat stress on the liver metabolism functions in *Huso dauricus*. In this study, we set one control group (19 °C) and four high-temperature treatment groups (22 °C, 25 °C, 28 °C, 31 °C) with 40 fish in each group for continuous 53-day heat exposure. Histological analysis, biochemical detection, and transcriptome technology were used to explore the effects of heat stress on the liver structure and functions of juvenile *Huso dauricus*. It suggested heat-stress-induced obvious liver injury and reactive oxygen species accumulation in *Huso dauricus* with a time/temperature-dependent manner. Serum total protein, transaminase, and alkaline phosphatase activities showed significant changes under heat stress (*p* < 0.05). In addition, 6433 differentially expressed genes (DEGs) were identified based on the RNA-seq project. Gene Ontology enrichment analysis showed that various DEGs could be mapped to the lipid-metabolism-related terms. KEGG enrichment and immunohistochemistry analysis showed that ferroptosis and FoxO signaling pathways were significantly enriched (*p* < 0.05). These results demonstrated that thermal stress induced oxidative stress damage in the liver of juvenile *Huso dauricus*, which triggered lipid metabolism disorder and hepatocyte ferroptosis to disrupt normal liver functions. In conclusion, chronic thermal stress can cause antioxidant capacity imbalance in the liver of *Huso dauricus* to mediate the ferroptosis process, which would finally disturb the lipid metabolism homeostasis. In further research, it will be necessary to verify the detailed cellular signaling pathways that are involved in the heat-stress-induced liver function disorder response based on the in vitro experiment, while the multi-organ crosswalk mode under the thermal stress status is also essential for understanding the comprehensive mechanism of heat-stress-mediated negative effects on fish species.

## 1. Introduction

*Huso dauricus* belongs to *Huso* genus of the Acipenseridae family and is mainly distributed in the Heilongjiang River basin and its tributaries in China [1]. As the oldest non-bony fish, *Huso dauricus* is known as a “living fossil in water” [2] and has extremely high ecological value [3]. Furthermore, its economic value is also worthy of attention because the caviar obtained from it is expensive and delicious. With the increased trend of market demand, the culture scale of *Huso dauricus* has been expanding, and thus the culture area has gradually expanded to lower latitudes to reduce production costs. However, *Huso dauricus* would face higher culture temperatures in these conditions. Because fish can be classified as ectotherms animals, temperature is an important abiotic factor that affects the survival, growth, and development of fish species [4]. Heat stress in fish can trigger a series of physiological responses, which can affect the normal functions of various organs [5,6]. So far, the sturgeon culture industry has expanded rapidly in other countries and continents. Therefore, chronic thermal stress caused by global climate warming and strong sunlight in summer might be one problem for the stable economic development of the sturgeon breeding industry worldwide. Since the 21st century, the availability of public genomic or transcriptomic data on sturgeon species has facilitated research progress on the detailed mechanism of heat-stress-induced tissue injury [7,8]. Yang et al. [9] executed transcriptome sequencing to reveal that heat stress could induce gill tissue damage in Siberian sturgeon (*Acipenser baerii*) and affect the osmoregulatory function of the gills. Other studies have shown that heat stress significantly damaged the intestines of sturgeon and disrupted the intestinal microbiota homeostasis [10]. However, studies on the detailed mechanisms of the negative effects of heat stress on *Huso dauricus* are relatively scarce [11,12].

The liver, as one major metabolism organ and the central hub of the detoxification process, is particularly vulnerable under heat stress conditions [13]. Oxidative stress is regarded as one critical mechanism in liver injury induced by thermal injury. Studies have shown that heat stress causes inflammation damage in the liver tissue of Chinese sturgeon (*Acipenser dabryanus*) by elevating the levels of liver oxidative stress biomarkers [14]. Research on zebrafish (*Danio rerio*) has also confirmed that the excessive reactive oxygen species (ROS) can aggravate the heptic oxidative stress response to attack the lipids, proteins, and DNA in hepatocytes and cause liver cell necrosis and apoptosis, which ultimately results in liver tissue damage [15].

Currently, research on *Huso dauricus* has mainly focused on wild resource surveys and protection [3,16,17] as well as artificial breeding technique development [18]. This study aims to explore the effects of heat stress on the liver structure and functions of *Huso dauricus*. Therefore, juvenile *Huso dauricus* individuals were exposed to a controllable high-temperature environment and then hepatic histopathological observation, biochemical parameters, transcriptome sequencing, and immunohistochemistry technology were performed. It may provide new insights into the molecular mechanism on the negative effects of thermal stress on sturgeons, which could promote the development of the aquaculture industry. In parallel, it can also provide a scientific basis for the protection of the wild population of *Huso dauricus*.

## 2. Materials and Methods

### 2.1. Overview of the Experimental Conditions

Juvenile *Huso dauricus* was collected from the Hulan Experimental Station of the Heilongjiang River Fishery Research Institute (Harbin, China). Before the experiment, 200 healthy *Huso dauricus* (body weight: 24 ± 2.5 g, body length: 15 ± 2.5 cm) were placed into an automatic temperature-controlled aquaculture system (100 cm × 60 cm × 60 cm). During the 2-week adaptation period, the culture conditions were maintained as follows: light–dark ratio = 12 h/12 h, water temperature = 19 ± 0.5 °C, pH = 7.0–8.5, dissolved oxygen ≥ 6 mg/L, and NH_4^+^_ concentration ≤ 0.005 mg/L. A commercial juvenile fish microbound diet (Shengsuo, China) feed was fed twice a day (Table 1), and the daily feeding amount was 3% of the juvenile body weight. The experiment was divided into five temperature groups (19 °C, 22 °C, 25 °C, 28 °C, and 31 °C) with 40 fish randomly selected in each group, and the 19 °C group was used as the control group, because this is the optimal breeding temperature for sturgeon species. And the water temperature of the other four groups was increased to the target temperature from 19 °C at a rate of 1 °C/h. The culture experiment lasted for 53 days. Individual survival number was recorded daily, and samples were collected on the 5th, 12th, 26th, and 53th days (D).

### 2.2. Sample Collection

All animal experiments in the present investigation were in accordance with the guidelines of the Laboratory Animal Ethics Committee of the Heilongjiang River Fishery Research Institute (Approval number: 20240909-001) and Directive 2010/63/EU for animal experiments. Before formal sampling, the test fish were fasted for at least 16 h. Nine fish were randomly selected from each group and anesthetized using MS-222 (40 mg/L), and the body length and weight were also measured. Blood samples were collected from the tail vein using sterile vessels with anticoagulant and then centrifuged for 28 min at 4 °C, and the serum was obtained for further biochemical index detection. Liver tissues were collected immediately after blood collection and washed with cold physiological saline two times; one part was fixed in Bouin’s fixative, and the other part was frozen in liquid nitrogen for 2 h and then transferred to an −80 °C environment.

### 2.3. Biochemical Indicators Detection

After the whole blood samples were collected into aseptic anticoagulant tubes with 1% heparin sodium, they were stored in a 4 °C environment and then transferred to the professional clinical laboratory. Subsequently, the whole blood samples were centrifuged at 11,500 r/min to obtain the serum and a series of biochemical parameters such as total protein (TP) content, alanine aminotransferase (ALT), activityaspartate aminotransferase (AST), and alkaline phosphatase (ALP) activities in serum were measured using the automatic biochemical analyzer (AU5800, Beckman Coulter, San Jose, CA, USA). In order to avoid the degradation of serum enzyme activities, all procedures above were executed within 1.5 h. Meanwhile, each serum sample was divided in triplicate to ensure the accuracy and robustness of the experimental data.

### 2.4. Hepatic ROS Fluorescence Staining and Fat Acculation Observation

Fresh liver tissues of juvenile *Huso dauricus* at 53D were fixed in Bouin’s solution for 48 h, and then they were dehydrated with alcohol, vitrified with xylene, and embedded in paraffin. Subsequently the liver tissues were sectioned, dried, stained with hematoxylin–eosin, and sealed with neutral resin to make paraffin sections. The histological structure was observed by optical microscopy (Eclipse Ci-L, Nikon, Tokyo, Japan).

Fresh liver tissue of juvenile *Huso dauricus* at 26 D and 53D were made into frozen sections under freezing conditions using Cryotome E microtome (Cryostar NX50, Thermo Fisher Scientific, Waltham, MA, USA). Frozen sections were rewarmed to room temperature and dried to remove the moisture. Subsequently, the hydrophobic isolation area surrounding the tissue was built using a liquid blocker super PAP pen (P0139, Beyotime, Shanghai, China) to prevent the liquid reagent from flowing away. The tissue autofluorescence quencher (P0122, Beyotime, Shanghai, China) was added to the tissue, allowing them to have full contact for 8 min, and the mixture was washed continuously with deionized water for 5 min. ROS staining solution (D7008, Sigma, Darmstadt, Germany) was added into the hepatic frozen sections, and the mixture was incubated at 37 °C in dark for 35 min. Then, the sections were washed 2 times with pre-cooled phosphate buffer solution (PBS), and following this, the sections were sealed with antifade mounting medium (P0131, Beyotime, Shanghai, China). Photographs were taken using a standing fluorescence microscope (Eclipse C1, Nikon, Tokyo, Japan). Nuclei were blue at a 330 nm excitation wavelength while the positive ROS area was stained red. The relative positive rate of each image was measured using Image J 1.51K software.

Hepatic frozen sections were also stained with oil red O dye solution (G1015, Servicebio, Wuhan, China). After this, the slices were differentiated and stained with hematoxylin solution (G1004, Servicebio, Wuhan, China). Finally, the slides were sealed with glycerin gelatin sealing adhesive (G1402, Servicebio, Wuhan, China) and then examined using a microscope. Under optical microscopy, the lipid droplets were orange-red to bright red, and nuclei were blue. The relative positive rate of each image was measured using Image J 1.51K software.

### 2.5. Hepatic Immunohistochemistry Analysis

Hepatic cyclooxygenase-2 (COX2), glutathione peroxidase 4 (GPX4), and Forkhead-box O 1 (FOXO1) expression levels were quantitatively analyzed using the immunohistochemistry technique. Hepatic frozen sections were dried at room temperature and then baked in an oven at 37 °C. After antigen repair, the slices were treated with 3% hydrogen peroxide solution (Angergech, Shandong, China). An isolation area surrounding the tissue was formed with a liquid blocker super PAP pen. After immersion in 3% bovine serum albumin solution (GC305010, Servicebio, Wuhan, China) and sealing at room temperature for 45 min, the slides were incubated with rabbit-source primary antibody (1:650 dilution, GPX4: GB114327; COX2: GB11077; FOXO1: GB114935, Servicebio, Wuhan, China) and horseradish-peroxidase-conjugated goat anti-rabbit IgG (dilute at 1;500, G1215, Servicebio, Wuhan, China) successively. The freshly prepared AEC color developing solution (A2010, Solaibao Technology, Beijing, China) was added to the tissue for color development, and then the cell nuclei were re-stained with hematoxylin solution. Finally, the slides were sealed with glycerin gelatin sealing adhesive and then examined using a white light microscope (E100, Nikon, Tokyo, Japan). Nuclei stained with hematoxylin were blue, while the positive expression of the target protein was brown. The relative positive rate of each image was measured using Image J 1.51K software.

### 2.6. RNA Extraction, Library Construction, and Sequencing

Total RNA was extracted from liver tissue samples using TRIzol reagent (15596018, Thermo Fisher Scientific, USA) according to the manufacturer’s instructions. RNA integrity was evaluated using the Agilent 2100 Bioanalyzer (Agilent Technologies, Santa Clara, CA, USA). The samples with RNA Integrity Number (RIN) ≥ 7 were subjected to the subsequent analysis. The libraries were constructed using TruSeq Stranded mRNA LTSample Prep Kit (Illumina, San Diego, CA, USA) according to the manufacturer’s instructions. Then, these libraries were sequenced on the Illumina sequencing platform (HiSeqTM 2500), and 125 bp/150 bp paired-end reads were generated.

### 2.7. Sequence Assembly and Function Annotation Analysis

Raw data was output by the Illumina Hiseq^TM^ 2500 sequencing platform and stored as fastq format. Raw reads were filtered using Trimmomatic to remove adaptor and low-quality sequences. After quality control, the clean reads were spliced into contigs sequences based on the de novo assembly method in the Trinity tool. The longest transcript was chosen as Unigene based on the sequence similarity. Subsequently, DIAMOND 5.1 and HMMER 3.0 software were utilized to align the Unigene using Nonredundant (NR), SwissProt, Clusters of orthologous groups for eukaryotic complete genomes (KOG), Gene ontology (GO), Kyoto Encyclopedia of Genes and Genomes (KEGG), eggnog, and Pfam databases and then used to obtain the gene function annotation. Meanwhile, the functional analysis of Unigenes was performed in the database.

### 2.8. Differentially Expressed Gene (DEG) Identification and Enrichment Analysis

Fragments per kilobases per million mapped read (FPKM) values for each Unigene were calculated using eXpress software 1.5.1. The relative fold changes of DEGs were analyzed by standardizing the genes in each treatment group based on DESeq 2 software. *p* value < 0.05 and |log_2_ (Fold Change)| > 1 was regarded as the threshold for significantly differential expression. Meanwhile, GO and KEGG enrichment analysis were performed to identify the gene functions and involved cellular signaling pathways of DEGs.

### 2.9. Statistical Analysis

SPSS 26.0 (IBM, New York, NY, USA) software was used for the statistical analysis process of experimental data. The normal distribution test for original experiment data was performed based on the Shapiro–Wilk method, and then Levene’s test was used to verify whether the data had homogeneity of variance. One-way analysis of variance (ANOVA) and Duncan’s post hoc test were utilized to verify the difference significance between groups. Data were expressed as mean± standard deviation, and *p* < 0.05 was considered as the threshold value of inter-group significance. Microsoft Office Excel 2016 and Graphpad Prism 9 software were selected as graphics programs for the data visualization. The bioinformatics analysis process for the transcriptome project was implemented by Lian Chuan-Biotechnology Co., Ltd. (Hangzhou, China).

## 3. Results

### 3.1. Growth Performance and Survival Rate of Juvenile Huso dauricus Under Heat Stress

**Figure 1:**
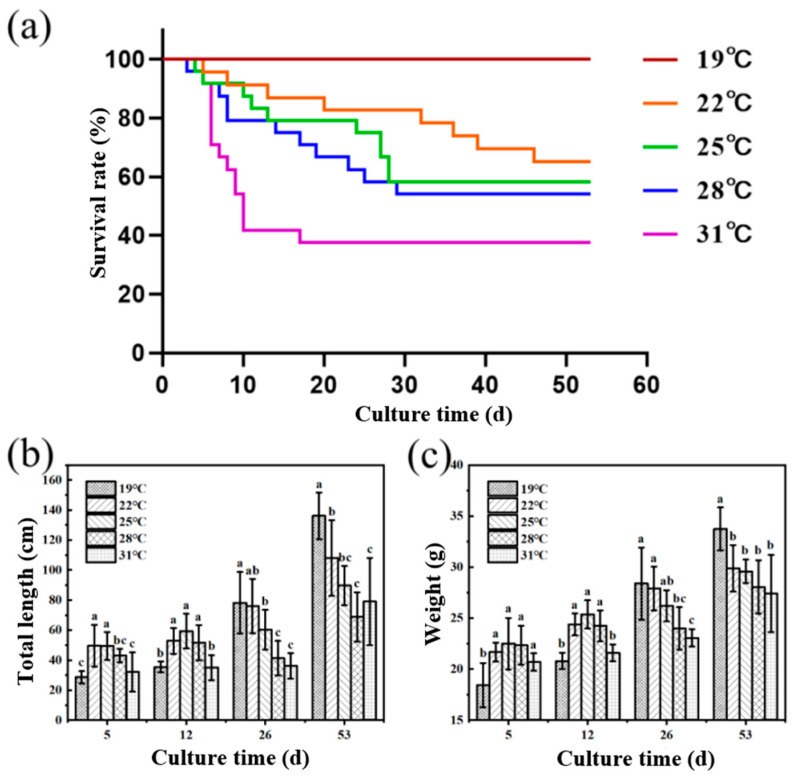
Effects of heat stress on growth performance and survival rate of juvenile *Huso dauricus* (n = 9). (**a**) Effects of heat stress on survival rate of juvenile *Huso dauricus*. (**b**) Effects of heat stress on total length of juvenile *Huso dauricus*. (**c**) Effects of heat stress on weight of juvenile *Huso dauricus*. Data are mean ± standard deviation. Different letters represent the significant difference between four groups (*p* < 0.05). One-way ANOVA was utilized to verify the difference significance between groups.

As shown in Figure 1, the survival rate of juvenile *Huso dauricus* decreased with the elevated breeding temperature. The survival rates of juvenile *Huso dauricus* on 53 D were 100% (19 °C), 66.67% (22 °C), 58.33% (25 °C), 56.25% (28 °C), and 37.5% (31 °C), respectively (Figure 1a). On 5 D and 12 D, the body weights of juvenile *Huso dauricus* in 22 °C and 25 °C groups were significantly higher than that in the 19 °C group, and there was no significant difference between the 31 °C and 19 °C groups (*p* < 0.05). Overall, this shows a trend of increasing first and then decreasing (Figure 1b). On 26 D and 53 D, the body weight of juvenile *Huso dauricus* decreased with the increased culture temperature, and that in the 31 °C group was significantly lower than that in the 19 °C group (*p* < 0.05) (Figure 1c).

### 3.2. Pathological Changes of Liver Tissue in Huso dauricus Under Heat Stress

**Figure 2:**
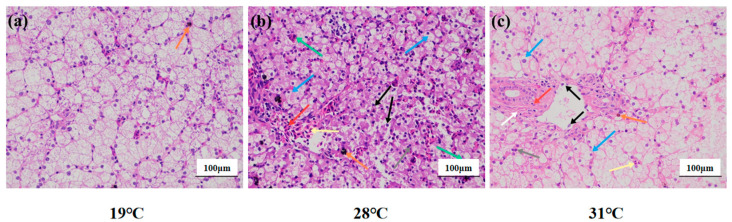
Liver structure of juvenile *Huso dauricus* under heat stress (HE × 200). (**a**) Liver structure of the 19 °C group of juvenile *Huso dauricus*. (**b**) Liver structure of the 28 °C group of juvenile *Huso dauricus*. (**c**) Liver structure of the 31 °C group of juvenile *Huso dauricus*.

In the 19 °C group (Figure 2a), the liver tissue structure was basically complete and clear boundaries between cells; loose cytoplasm and clear nuclei also could be observed. Brownish yellow pigment deposits were occasionally seen in the stroma (orange arrow). In the 28 °C group, the hepatocytes were irregularly arranged, the boundaries between the cells were not clear, the hepatocytes were vacuolated (blue arrow), and the nuclei were shrunken (gray arrow). Occasionally, the cytoplasm of hepatocytes was disintegrated while cell nuclei were naked (black arrow). In parallel, the number of red cells in the hepatic sinusoids was abundant (yellow arrow), while the nuclei were different in size (green arrow) and the blood vessels were congested (red arrow). Surprisingly, a small amount of black-brown material was deposited in the stroma (orange arrows) (Figure 2b). In the 31 °C group, the hepatocytes were arranged irregularly, the cells were highly swollen, and the intercellular boundaries were disorderly. Meanwhile, numerous hepatocytes were vacuolated (blue arrow), while shrunken nuclei (gray arrow) were also observed. Occasionally, the cytoplasm of hepatocytes was disintegrated, and the nucleus was bare (black arrow). The hepatic sinusoids were not clear, and occasional red blood cells were found in the sinusoids (yellow arrows). Connective tissue hyperplasia (white arrows), inflammatory cell infiltration (red arrows), and brown-yellow pigment deposition (orange arrows) were occasionally seen around the bile duct (Figure 2c).

### 3.3. Effects of Heat Stress on Serum Liver Function Indexes of Juvenile Huso dauricus

**Figure 3:**
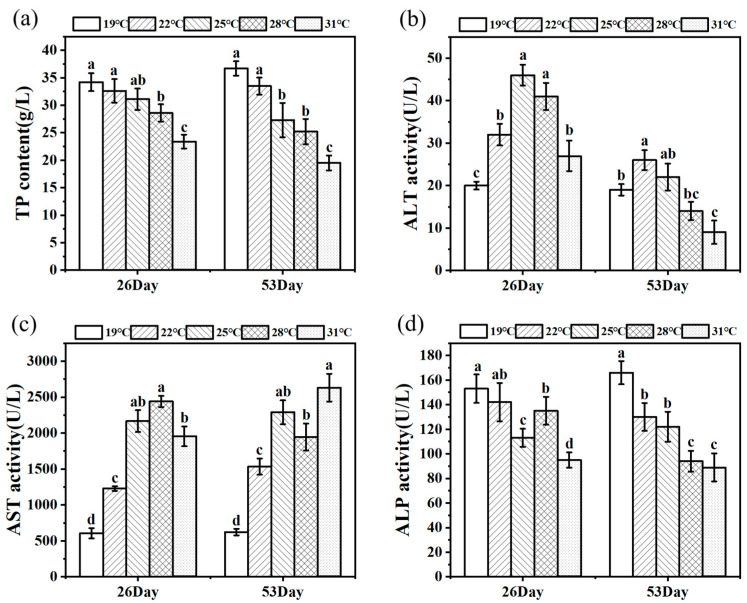
TP level (**a**) and ALT (**b**), AST (**c**), and ALP (**d**) activity in serum under thermal stress (n = 9). Data are described as mean ± standard deviation. Different letters represent the significant difference between four groups (*p* < 0.05). One-way ANOVA was utilized to verify the difference significance between groups.

Figure 3 shows the change trends of TP content and ALT, AST, and ALP activities of juvenile *Huso dauricus* on 26 D and 53 D. Serum TP content decreased with the increased culture temperature, and the TP content in the 31 °C group was significantly lower than that of other temperature groups (*p* < 0.05) (Figure 3a). On 26 D, ALT activity under heat stress was significantly higher than that in the 19 °C group (*p* < 0.05). 

In parallel, on 53 D, ALT activity in the 22 °C group was markedly higher than that in the other three groups except for the 25 °C group (*p* < 0.05), while the 31 °C group had the lowest ALT activity and was significantly lower than the control group (*p* < 0.05) (Figure 3b). Meanwhile, AST activities in heat stress groups were significantly higher than that in the 19 °C group (*p* < 0.05). On 26 D, ALP activity in the 31 °C group was significantly lower than that in the other three groups (*p* < 0.05). As expected, ALP activity in serum was significantly decreased under heat stress conditions compared to the 19 °C group (*p* < 0.05, Figure 3d).

### 3.4. ROS and Fat Content in the Liver Tissue of Juvenile Huso dauricus Under Heat Stress

**Figure 4:**
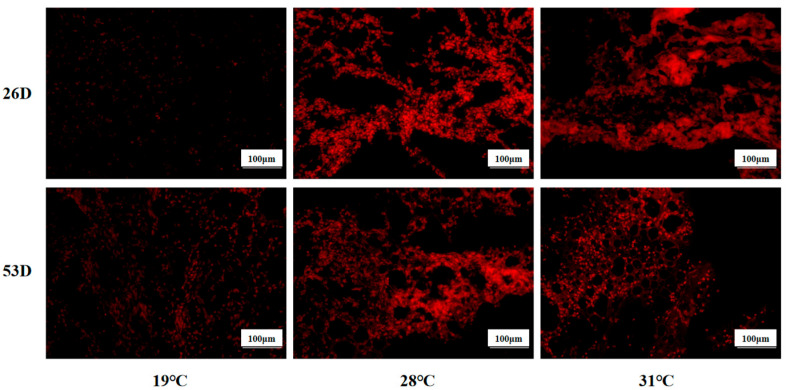
ROS levels in liver tissue in juvenile *Huso dauricus* (200×). Red represents positive ROS area.

ROS levels in liver tissue of juvenile *Huso dauricus* under heat stress are shown in Figure 4. It is shown that the ROS level in the 28 °C and 31 °C groups was significantly higher than in the 19 °C group on 26 D and 53 D (*p* < 0.05).

**Figure 5:**
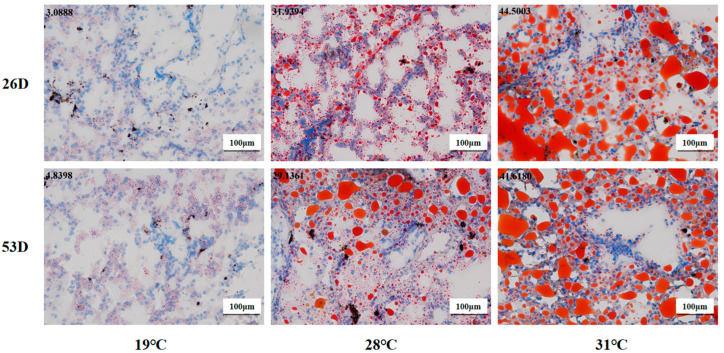
Fat content in liver tissue in juvenile *Huso dauricus* (200×). Red represents hepatic fat and blue represents nuclei. Data at the top-left corner represent the positive ratio of hepatic fat.

As shown in Figure 5, the fat content of liver tissue in the 28 °C and 31 °C groups was obviously elevated compared to the 19 °C group on 26 D and 53 D (*p* < 0.05). Our results suggest that heat stress could cause ROS and fat accumulation in the liver tissue of juvenile *Huso dauricus* with a temperature-dependent manner.

### 3.5. Sequencing Data Assembly

A total of 65.85 GB raw reads were obtained in the present transcriptome project. After filtering the low-quality, redundant, and adaptor sequences, the average GC content and valid rate of each sample were 47.61% and 94.16%, respectively, while the Q30 base distribution ranged from 93.59% to 94.76% (Table 2). Our results suggest that the base separation phenomenon was not observed in the hepatic transcript of *Huso dauricus*. These results indicate that the sequencing data were reliable and could be used for further analysis.

### 3.6. Transcriptome Annotation

**Figure 6:**
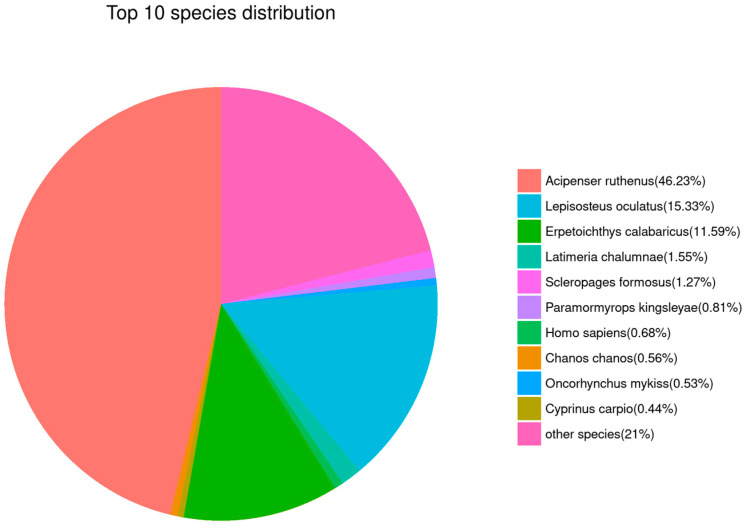
Gene annotation analysis based on the NR database. Sector areas with different colors represent the relative annotation ratio of each species.

The results of sequence alignment for all Unigenes based on public databases are shown in Table 3. The top five species with similar sequences were *Acipenser ruthenus* (46.23%), *Lepisosteus oculatus* (15.33%), *Erpetoichthys calabaricus* (11.59%), *Latimeria chalumnae* (1.55%), and *Scleropages formosus* (1.27%, Figure 6). Obviously, this suggests that Unigene sequences in our de novo transcriptome results could be mainly mapped to the genes that belong to sturgeon.

### 3.7. Summary for DEGs

**Figure 7:**
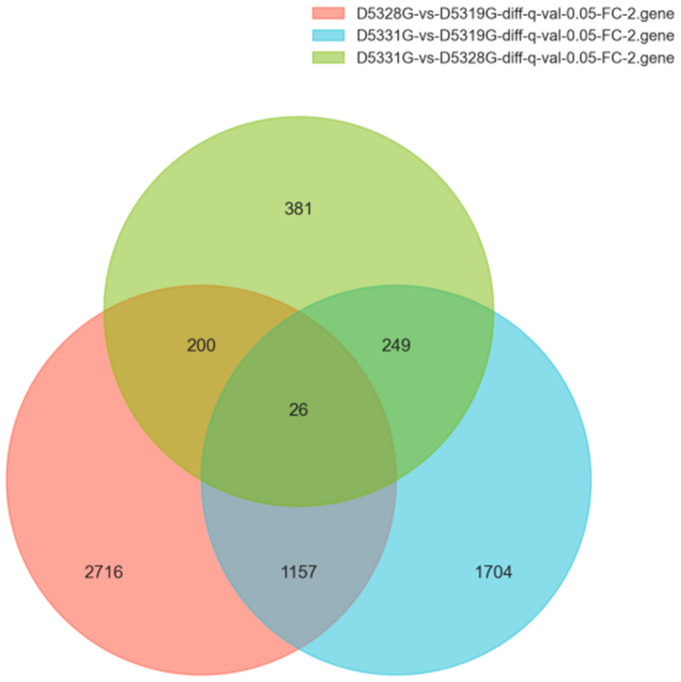
Venn diagrams of DEGs in various group combinations: D5328G vs. D5319G, D5331G vs. D5319G, and D5331G vs. D5328G.

**Figure 8:**
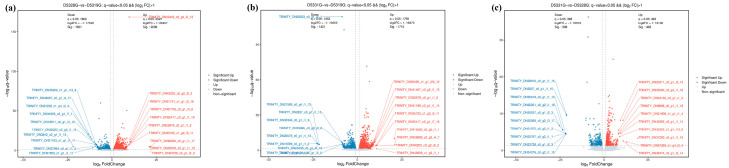
Volcano map of DEGs in D5328G vs D5319G (**a**), D5331G vs D5319G group (**b**) and D5331G vs D5328G group (**c**). Red indicates significant up, blue indicates significant down, and grey indicates non-significant. The horizontal coordinate is log_10_ q-value, while the vertical coordinate is log_2_ fold change.

**Figure 9:**
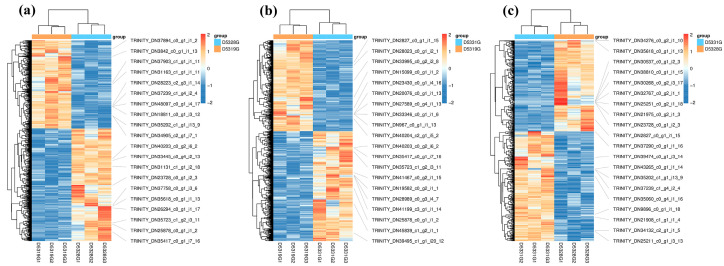
Heatmap of DEGs in D5328G vs D5319G (**a**), D5331G vs D5319G group (**b**) and D5331G vs D5328G group (**c**). The color of each module indicates the log2 fold change value of each DEG. The color scale ranging from red to blue indicates a decrease in gene expression.

In the current study, the DEG filtration threshold was set as the false discovery rate (FDR) value < 0.05 and |log2 (Fold Change)| > 1. A total of 6433 DEGs were identified among the 19 °C, 28 °C, and 31 °C groups (Figure 7). It was shown that there were 4099 DEGs between 28 °C and 19 °C, among which 2298 were upregulated and 1801 were downregulated (Figure 8a and Figure 9a), and there were 3136 DEGs between 31 °C and 19 °C, among which 1715 were upregulated and 1421 were downregulated (Figure 8b and Figure 9b). Additionally, A total of 856 DEGs were identified between 31 °C and 28 °C, among which 468 were upregulated and 388 were downregulated (Figure 8c and Figure 9c).

### 3.8. GO Enrichment Analysis for DEGs

**Figure 10:**
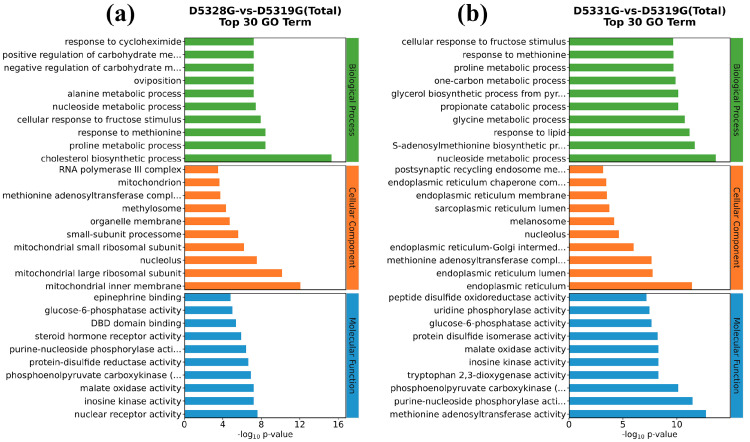
GO enrichment for hepatic DEGs in D5328G vs D5319G (**a**) and D5331G vs D5319G group (**b**). The top 10 terms for BP, CC, and MF are displayed in the diagram.

In the 28 °C vs. 19 °C group, the top five GO terms that were enriched in the biological process (BP) were the cholesterol biosynthetic process, proline metabolic process, response to methionine, cellular response to fructose stimulus, and nucleoside metabolic process. Meanwhile, the top five GO terms enriched in the cellular component (CC) were mitochondrial inner membrane, mitochondrial large ribosomal subunit, nucleolus, mitochondrial small ribosomal subunit, and small-subunit processome. In addition, the top five GO terms enriched in the molecular function (MF) were nuclear receptor activity, inosine kinase activity, malate oxidase activity, phosphoenolpyruvate carboxykinase (GTP) activity, and protein-disulfide reductase activity (Figure 10a).

Compared to the 28 °C vs. 19 °C group, the top five GO terms that belonged to BP terms in the 31 °C vs. 19 °C group also included the nucleoside metabolic process, while the top five GO terms enriched in the CC terms also included nucleolus. Interestingly, the top five GO terms enriched in the MF terms also included phosphoenolpyruvate carboxykinase (GTP) activity and inosine kinase activity (Figure 10b).

**Figure 11:**
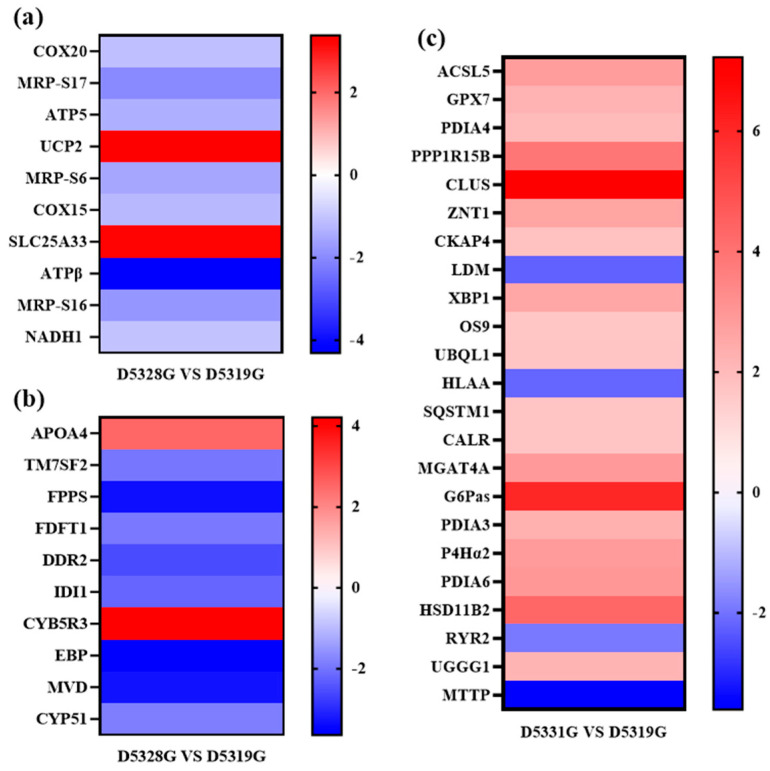
Heat map for typical DEGs that were enriched in the mitochondrial inner membrane (**a**), cholesterol biosynthetic process (**b**), and endoplasmic reticulum (**c**) GO terms. Colors indicate the log2 fold change value of each DEG. The color scale ranging from red to blue indicates a decrease in gene expression.

Typical DEGs from the mitochondrial inner membrane, cholesterol biosynthetic process, and endoplasmic reticulum were filtered based on the order in which the *p* value increased (Figure 11).

### 3.9. KEGG Enrichment Analysis for DEGs

In the 28 °C vs. 19 °C group and 31 °C vs. 19 °C group, all DEGs could be significantly enriched in 22 and 33 KEGG pathways, respectively. Among them, it was found that ferroptosis and the FoxO signaling pathway were significantly enriched in both groups.

**Figure 12:**
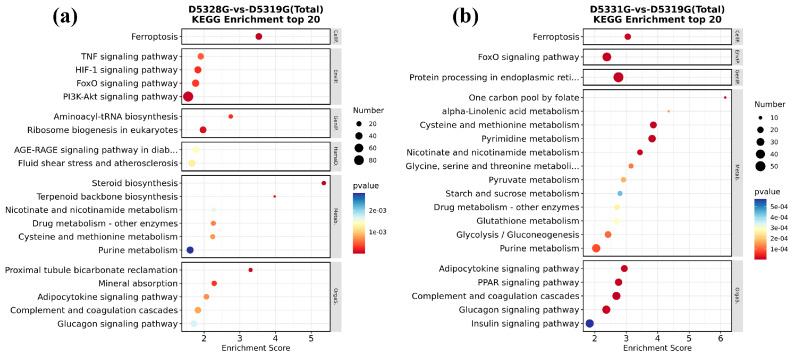
KEGG significant enrichment results in the top 20 in D5328G vs D5319G (**a**) and D5331G vs D5319G group (**b**). The horizontal coordinate was enrichment score, while the vertical coordinate was the names of the pathways that were significantly enriched. The color of each dot represents the corrected *p*-value of the corresponding item. The color scale ranging from red to blue indicates a decrease in gene expression.

In addition, DEGs in the 28 °C vs. 19 °C group were mainly enriched in the TNF signaling pathway; HIF-1 signaling pathway; PI3K-Akt signaling pathway; aminoacyl-tRNA biosynthesis; ribosome biogenesis in eukaryotes; proximal tubule bicarbonate reclamation; and mineral absorption, etc. (Figure 12a). In the 31 °C vs. 19 °C group, DEGs were also mainly enriched in protein processing in the endoplasmic reticulum; one carbon pool by folate; alpha-linolenic acid metabolism; cysteine and methionine metabolism; pyrimidine metabolism; adipocytokine signaling pathway; PPAR signaling pathway; complement and coagulation cascades; and glucagon signaling pathway, etc. (Figure 12b). These pathways are mainly involved in processes such as energy metabolism, cell proliferation, and information transmission.

### 3.10. Expression Levels of Ferroptosis/FoxO-Signaling-Pathway-Related Proteins

**Figure 13:**
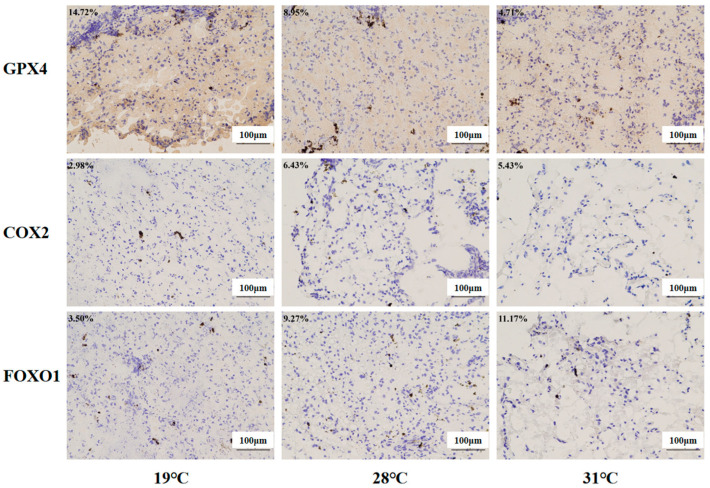
Immunohistochemistry analysis of liver tissue in juvenile Huso dauricus (200×). Each data point in the figure represents the positive area of target proteins. Blue indicates nuclei, and brown indicates the positive expression of the target protein area.

Immunohistochemistry was used to investigate the protein expression levels of the ferroptosis regulators (COX2 and GPX4) and the FoxO signaling pathway regulators (FOXO1, Figure 13). Compared with the control group, significantly higher levels of COX2 and FOXO1 were found in the 28 °C group and 31 °C group (*p* < 0.05), while Gpx4 protein expression was inhibited markedly in these two groups (*p* < 0.05).

## 4. Discussion

### 4.1. Chronic Heat Stress Caused Hepatic Damage

The liver is an important organ for substance metabolism and immune defense in fish [19]. Changes in liver tissue structure and functions can be regarded as early indicators for external environmental stress [20,21]. Studies have shown that high temperature exposure could cause liver damage in *Micropterus salmoides* [5]. In the present study, the liver tissue showed vacuolar degeneration and exposed nucleus under chronic heat stress, indicating that high temperature would disrupt the normal liver tissue structure in juvenile *Huso dauricus*. It is consistent with the results that were found in *Acipenser dabryanus* under heat stress conditions [14].

### 4.2. Chronic Heat Stress Disrupted the Biochemical Parameter Homeostasis in Serum

TP is mainly synthesized by hepatocytes. When the liver is damaged, the hepatic synthesis function for proteins is inhibited. Dagoudo et al. [22] showed that the TP content of hybrid yellow catfish (*Pelteobagrus fulvidraco* × *P. vachelli*) decreased significantly under heat treatment. In this experiment, *Huso dauricus* serum TP content decreased significantly with the increase in stress temperature, which might be attributed to liver functions dysfunction caused by heat stress, thereby blocking the normal protein synthesis process. AST and ALT are mainly distributed in hepatocytes and display a low level in serum under normal physiological activities. When the liver function is damaged, AST and ALT in hepatocytes are released into the blood, causing serum concentration rise. Therefore, the levels of AST and ALT in serum can also reflect the liver damage degree [23,24,25]. In the present study, AST and ALT activities in the serum of juvenile *Huso dauricus* increased significantly with increased temperature. It was indicated that liver damage had occurred under high-temperature stress. A similar situation has been observed in other studies, in which the serum AST and ALT levels of *Clarias gariepinus* were significantly increased under heat stress [26]. ALP plays an important role in the body’s phosphorus metabolism. In this study, the serum ALP activity of juvenile *Huso dauricus* decreased with increased culture temperature, which could be attributed to phosphorus metabolism imbalance caused by long-term heat stress. Similar results also were found in heat stress studies in *Takifugu obscurus* [27]. Taken together, it was inferred that *Huso dauricus* would initiate the compensatory physiological response mechanism to resist the negative effects of chronic thermal stress on the liver functions of sturgeon. Meanwhile, alkaline phosphatase activity usually is elevated with the occurrence of tissue injury. However, its synthesis process could also be inhibited under the extremely high temperature and liver tissue injury induced by a prolonged heat exposure period, which also would disrupt the hepatic synthesis capacity for alkaline phosphatase.

### 4.3. Chronic Heat Stress Induced the Energy Metabolism Unequilibrium

Mitochondria are the main site of ROS formation, and the impairment of mitochondrial functions would lead to the excessive accumulation of cellular ROS [28,29,30]. In turn, excessive generation of ROS would aggravate mitochondrial damage to inhibit the normal oxidative phosphorylation function and cause cellular energy metabolism disorder. In this study, it can be observed that the hepatic ROS level in the high-temperature treatment group was higher than that in the control group, indicating that heat stress accelerated the ROS accumulation in the liver, which might lead to liver mitochondria damage. Normal liver functions depend on the energy supplement provided by mitochondria. GO term enrichment analysis also showed that DEGs (such as Cytochrome c oxidase protein 20 (*COX20*); ATP synthase-coupling factor 6, mitochondrial *(ATP5J*); and ATPβ synthase subunit beta, mitochondrial (*ATP5B*)) could be significantly mapped in the mitochondrial inner membrane. It was speculated that the *Huso dauricus* mitochondria function imbalance caused by heat stress would affect the normal energy metabolism process in the liver.

### 4.4. Chronic Heat Stress Induced the Lipid Metabolism Disturbance

Cholesterol is one lipid substance and is mainly synthesized in the liver [31]. When liver function is impaired, it results in the disorder of the cholesterol metabolism process. Isopentenyl-diphosphate delta-isomerase 1 (*IDI1*) encodes an enzyme that catalyzes the interconversion of isopentenyl-diphosphate (IPP) to dimethylallyl pyrophosphate (DMAPP) [32]. Subsequently, farnesyl pyrophosphate synthase (FPPS) can catalyze the production of farnesyl pyrophosphate (FPP), which is an important intermediate in the biosynthesis process of cholesterol [33]. Moreover, farnesyl-diphosphate farnesyltransferase 1 (FDFT1) is one critical enzyme in cholesterol synthesis. FDFT1 catalyzes the conversion of FPP to squalene, which is regarded as an early step in the cholesterol synthesis process. Studies have shown that the expression of FDFT1 was significantly increased in Sprague-Dawley (SD) rats with acute hypoxia injury, and the lipid metabolism pathway was abnormally activated [34], indicating that exogenous stress could affect the expression of FDFT1 and then affect the synthesis of lipid substances. Gonzalez-Rivera et al. [35] demonstrated that FDFT1 transcript is susceptible to an oxidation effect induced by an oxidative stress response in the cholesterol biosynthesis pathway, resulting in decreased transcription expression of FDFT1. In addition, knockdown of FDFT1 could also lead to the reduction of endogenous cholesterol content [36]. In the present study, the expression of *IDI1*, *FPPS*, and FDFT1 was significantly downregulated in the 28 °C group on 53 D, suggesting that heat stress might inhibit the synthesis of FPP by inhibiting the expression of various genes that were involved in the cholesterol synthesis. In addition, mRNA transcribed by the FDFT1 gene might be oxidized due to hepatic oxidative stress reaction, which would lead to disorder in the cholesterol synthesis pathway. Apolipoprotein A-IV (*APOA4*) was also enriched in the cholesterol synthesis term. As one result of the present study, the APOA4 protein was upregulated. Studies have shown that APOA4 is involved in lipid storage [37] and the process of reverse cholesterol transport [38]. Based on the oil red O staining results, it could be found that the hepatic fat contents in the high-temperature groups were higher than the 19 °C group, indicating that heat stress would lead to excessive fat accumulation in the liver to disrupt the normal lipid metabolism.

Cholesterol synthesis is an energy-consuming process that requires abundant ATP synthesis [39]. COX20, which is known as FAM36A, is one critical factor in the assembly process of mitochondrial cytochrome c oxidase and respiratory chain complex IV [40]. It was reported that the mitochondrial complex IV level was decreased in patients with the *COX20* gene mutation [41]. Dong et al. [42] found that knockdown of *COX20* expression would block the assembly of complex IV, thereby affecting the respiratory capacity for cells. Mitochondrial respiratory chain complexes undergo an oxidative phosphorylation process in the inner mitochondrial membrane and use the electrochemical proton gradient to synthesize ATP [43]. In this process, ATP5J and ATP5B are involved in the synthesis of ATP [44]. In this study, the downregulation trends of *COX20*, *ATP5J*, and *ATP5B* expression levels demonstrated the abnormal mitochondrial respiratory chain functions and ATP synthesis disorder in *Huso dauricus* liver under heat stress, which would affect the normal energy metabolism in the liver. In addition, 28S ribosomal protein S17 (MRP-S17), 28S ribosomal protein S6 (MRP-S6), and 28S ribosomal protein S16 (MRP-S16) are mitochondrial ribosomal proteins that are involved in essential protein synthesis in mitochondria. The downregulation expression of these genes may affect the synthesis of critical enzymes and transporters on the mitochondrial inner membrane. It is also possible that these enzymes contain enzymes related to ATP synthesis. Our results suggested that heat stress might affect the assembly and function of mitochondrial complex IV by inhibiting the expression of COX20. On this basis, the expressions of the key factors ATP5J and ATP5B in the ATP synthesis process are inhibited, jointly affecting the oxidative phosphorylation process of mitochondria. This would lead to the obstruction of ATP synthesis. Meanwhile, heat stress may also affect the synthesis of key enzymes in the mitochondrial ATP synthesis process by inhibiting the expression of mitochondrial ribosomal proteins, thereby influencing ATP synthesis, leading to abnormal mitochondrial energy metabolism in hepatocytes and insufficient energy supply to hepatocytes, as well as further affecting hepatic lipid metabolism and cholesterol synthesis and other functions. The disorder in lipid metabolism in the liver also could lead to hepatocyte steatosis [45], which was consistent with the histopathological results of liver tissue in the present study.

### 4.5. FOXO1 Signaling Pathway Involved in the Heat-Stress-Induced Liver Function Imbalance

Furthermore, our investigation inferred that heat stress would promote FOXO1 expression in the liver. FOXO1 is an important transcription factor involved in the regulation of oxidative stress, apoptosis, and lipid metabolism [46,47]. A previous study found that [48] the activation of *FOXO1* gene expression is accompanied by the hepatic lipid deposition process. Liu et al. [49] also confirmed that chronic stress led to lipid deposition in the liver by activating *FOXO1*. Our results suggest that the upregulated expression trend of the *FOXO1* gene under heat stress might accelerate the liver fat deposition process in juvenile *Huso dauricus*. As expected, oil red O staining also demonstrated that chronic thermal stress would promote excessive fat deposition in the liver.

### 4.6. Chronic Heat Stress Caused Endoplasmic Reticulum Function Collapse

Increasing evidence has suggested that endoplasmic reticulum stress (ERS) might play a negative role in aquatic animals under high temperature stress [50,51]. Li et al. [52] showed that increased temperature caused significant changes in the expression levels of these genes related to ER protein processing in rainbow trout. Wang et al. [53] also found ER protein processing pathways were significantly enriched in the heat stress studies for larval *S. lucioperca*. Similarly, in this study, DEGs in the 31 °C vs. 19 °C groups were mainly enriched in the GO term related to “endoplasmic reticulum” and the “protein processing in endoplasmic reticulum” pathway. This indicated that the endoplasmic reticulum is involved in the response of beluga sturgeon to heat stress. The ER is an important organelle in eukaryotic cells, being responsible for protein synthesis, processing, transport, and lipid/carbohydrate synthesis [54]. When the body is stimulated by harsh environments such as high temperature and oxidative stress, the physiological functions of the ER will be disturbed to induce the accumulation of unfolded or misfolded proteins in the ER lumen, which would further aggravate ER dysfunction and trigger a series of cytoprotective mechanisms to maintain its homeostasis [55]. In response to ER stress, cells recognize misfolded and unfolded proteins through the unfolded protein response (UPR) and initiate endoplasmic-reticulum-associated degradation (ERAD). Subsequently, these proteins are transported into the cytoplasm and degraded through the ubiquitin–proteasome pathway or autophagy–lysosomal pathway [56].

UPR is a highly conserved adaptive protective response to stress in cells. As one important regulator in UPR, X-box-binding protein 1 (XBP1) is activated during ER stress and can upregulate calreticulin (CALR), protein disulfide-isomerase A3 (PDIA3), protein disulfide-isomerase A4 (PDIA4), and protein disulfide-isomerase A6 (PDIA6) [57,58,59,60] to enhance ER folding capacity and relieve ERS response. UDP-glucose:glycoprotein glucosyltransferase 1 (UGGG1) is involved in the glycosylation modification process in the endoplasmic reticulum and re-glycosylated misfolded proteins to facilitate their recognition by the ERAD pathway [61]. Both protein OS-9 (OS9) and ubiquilin-1 (UBQL1) recognize and transport misfolded proteins for proteasomal degradation [62,63]. In this study, the expressions of *XBP1*, *CALR*, *PDIA3*, *PDIA4*, *PDIA6*, *UGGG1*, *OS9*, and *UBQL1* were significantly upregulated in juvenile *Huso dauricus* under high-temperature stress. Similarly, in the study of *Haaliotis discus hannai* [64] and *Ctenopharyngodon idella* [65], significant upregulation trends of ER-stress-related genes were also found under stress conditions. Taken together, our results suggest that juvenile *Huso dauricus* could respond to the comprehensive effects of heat stress on hepatic functions by mediating the ERS reaction to maintain ER function homeostasis and ensure the normal protein synthesis process in the liver.

### 4.7. Chronic Heat Stress Mediated Ferroptosis in Hepatocytes

KEGG enrichment analysis also found that the “ferroptosis” pathway was significantly enriched in the 28 °C vs. 19 °C group and 31 °C vs. 19 °C group. Ferroptosis is an iron-dependent cell death mode and is characterized by intracellular ROS/lipid peroxide accumulation and the inhibition of GPX4 gene expression [66,67]. When the intracellular free iron level was increased due to exogenous environment stress sources, it can accelerate ROS production through the Fenton reaction and then attack membrane phospholipid polyunsaturated fatty acids (PUFAs) and cause lipid peroxidation and cell membrane structure destruction, which would finally trigger cell ferroptosis [68,69]. GPX4 could utilize glutathione (GSH) as a substrate to prevent the accumulation of cellular lipid peroxides and inhibit the occurrence of ferroptosis [70]. Studies have shown that heat stress causes ferroptosis in broiler hepatocytes [71]. Similarly, in the present study, heat stress promoted the ROS accumulation in the liver, which might contribute to the acceleration process of ferroptosis in the liver of juvenile *Huso dauricus* [72]. In addition, studies have shown that the inflammatory factor COX2 could be regarded as the accelerator factor for ferroptosis activation [73,74]. In the present study, it was found that GPX4 protein level was significantly decreased while COX2 protein level was significantly elevated under heat stress, which confirmed that thermal stress could cause the occurrence of an aseptic inflammation response in the liver of juvenile *Huso dauricus* by mediating oxidative stress injury, which would finally mediate the ferroptosis process to initiate the compensatory physiological response under heat stress conditions.

### 4.8. Advantage and Limitations

Our investigation utilized the high-throughput sequencing technique to overview the overall hepatic transcriptome profiling dynamic under the chronic heat stress status, which can be beneficial for understanding the critical injury mechanism on the negative effects of thermal stress on the liver of *Huso dauricus*. Nevertheless, the present study only identified the core metabolism pathways that might be affected by thermal stress based on bioinformatics methods. Therefore, it is essential to confirm that these differential signaling pathways could involve hepatic injury caused by heat stress using an in vitro experiment while multi-tissue research also could be performed to reveal the co-regulation mechanism of various organs in fish species under thermal stress.

## 5. Conclusions

Chronic heat stress triggered oxidative stress response in juvenile *Huso dauricus* to mediate hepatic lipid metabolism dysfunction and eventually induce hepatocyte ferroptosis and tissue injury. In addition, liver function disorder caused by chronic thermal stress in *Huso dauricus* displayed an obvious time/temperature-dependent manner. Based on the current results, it was shown that lipid metabolism disorder might be the essential mechanism of heat-stress-induced hepatic injury. Therefore, it was inferred that developing targeted lipid metabolism regulation agents might alleviate the negative effects of chronic thermal stress on the liver in sturgeon. Our research can contribute to providing basic data for exploring the detailed injury mechanism of chronic heat stress to *Huso dauricus*. In addition, our results also shed light on the healthy breeding for sturgeons and the wild population conservation biology. In addition, it was necessary to explore the comprehensive impacts of heat stress on the overall metabolism functions of sturgeon based on the molecular biology technology and identify the core signaling pathways to develop a bio-activator that could relieve the negative effects of heat stress on sturgeon species.

## Figures and Tables

**Table 1:** Basic feed ingredient composition.

Component	Contents (%)
Crude protein	≥43
Crude fat	≥8
Crude fibre	≤6
Crude ash	≤17
Calcium	≤5
Total phosphorus	≥1.0
Lysine	≥2.0
Moisture	≤12

**Table 2:** Summary for RNA-seq data.

Sample	Raw Reads (M)	Raw Bases (G)	Clean Reads (M)	Clean Bases (G)	Valid Bases (%)	Q30 (%)	GC (%)
D5319G1	49.04	7.36	48.27	6.95	94.54	93.93	47.02
D5319G2	48.13	7.22	47.38	6.81	94.35	93.59	47.24
D5319G3	47.50	7.13	46.79	6.69	93.84	94.48	47.04
D5328G1	48.74	7.31	48.03	6.91	94.46	94.06	47.10
D5328G2	51.33	7.70	50.52	7.25	94.12	94.16	47.79
D5328G3	47.62	7.14	46.87	6.73	94.26	94.11	48.01
D5331G1	48.03	7.20	47.28	6.76	93.89	94.74	48.48
D5331G2	48.97	7.35	48.19	6.90	93.89	94.60	47.54
D5331G3	49.61	7.44	48.88	7.00	94.06	94.76	48.30

**Table 3:** Annotation results for Unigenes based on a public database.

Database	Number of Notes	300 ≤ Length < 1000	Length ≥ 1000
NR	47,886 (43.61%)	19,432 (17.69%)	28,454 (25.91%)
Swissprot	38,140 (34.73%)	12,585 (11.46%)	25,555 (23.27%)
KEGG	15,570 (14.18%)	5268 (4.80%)	10,302 (9.38%)
KOG	27,149 (24.72%)	8420 (7.67%)	18,729 (17.05%)
eggNOG	40,794 (37.15%)	14,412 (13.12%)	26,382 (24.02%)
GO	33,749 (30.73%)	11,069 (10.08%)	22,680 (20.65%)
Pfam	29,707 (27.05%)	7023 (6.40%)	22,684 (20.66%)

## Data Availability

The original data used and generated in the present research project are available from the corresponding authors on request with a completed Data Transfer Agreement. Raw sequencing files have been uploaded to the public Gene Expression Omnibus database of the National Center for Biotechnology Information website.
